# Comprehensive analysis of IRF8-related genes and immune characteristics in lupus nephritis

**DOI:** 10.3389/fphar.2024.1468323

**Published:** 2024-12-09

**Authors:** Zhibin Yu, Chenghui Zheng, Yilun Wang

**Affiliations:** ^1^ Fuzong Clinical Medical College of Fujian Medical University, Fuzhou, Fujian, China; ^2^ Department of Dermatology, Huashan Hospital, Fudan University, Shanghai, China

**Keywords:** lupus nephritis, IRF8, biomarkers, drug-gene interaction, immune infiltration

## Abstract

**Background:**

There are currently no reliable diagnostic biomarkers or treatments for lupus nephritis (LN), a complication of systemic lupus erythematosus. Objective: We aimed to explore gene networks and potential biomarkers for LN by analyzing the GSE32591 and GSE113342 datasets from the Gene Expression Omnibus database, focusing on *IRF8* and *IRF8*-related genes.

**Methods:**

We used differential expression analysis, functional enrichment, protein-protein interaction (PPI) network construction, and the CIBERSORT algorithm for immune infiltration assessment. To validate the expression levels of the IRF8 gene in the kidneys of lupus mice models, we used quantitative real-time PCR (qRT-PCR) and Western blotting (WB). A diagnostic classifier was built using the RandomForest method to evaluate the diagnostic potential of selected key genes. To bridge our findings with potential therapeutic implications, we used the drug-gene interaction database to predict drugs targeting the identified genes.

**Results:**

Twenty co-differentially expressed genes (DEGs) were identified, with IRF8 exhibiting significant expression differences and potential as a biomarker. Functional enrichment analysis revealed pathways associated with immune response. Validation through qRT-PCR and WB confirmed that the IRF8 gene and its protein exhibited elevated expression levels in the kidneys of lupus mice compared to control groups. The diagnostic classifier revealed impressive accuracy in differentiating LN from control samples, achieving a notable area under the curve values across various datasets. Additionally, immune infiltration analysis indicated significant differences in the immune cell profiles between the LN and control groups.

**Conclusion:**

IRF8 and its related genes show promise as biomarkers and therapeutic targets for LN. These findings contribute to a deeper understanding of the molecular mechanisms involved in LN and may support the development of precision medicine strategies for improved patient outcomes.

## 1 Introduction

Systemic lupus erythematosus (SLE) is a chronic autoimmune inflammatory disease of unknown etiology, affecting approximately five million people worldwide (Kong et al., 2019; Udhaya Kumar et al., 2020). The female-to-male ratio among patients with SLE is approximately 7–9:1 (Kong et al., 2019). In patients with SLE, immune dysregulation leads to the production of autoantibodies targeting nuclear and cytoplasmic antigens. SLE also initiates autoimmune responses and inflammation across multiple organs, giving rise to a broad spectrum of clinical manifestations. Mild cases may be limited to skin involvement, such as erythema or oral ulcers, while severe cases can involve critical damage to the hematologic, renal, or nervous systems, potentially posing life-threatening risks. (Yap and Chan, 2019). LN is a manifestation of SLE that affects approximately 39% of patients (Kong et al., 2019) and is a major risk factor for morbidity and mortality (Almaani et al., 2017). LN is characterized by glomerulonephritis (Yang and Li, 2019). About 10% of all patients with LN develop end-stage renal disease (ESRD) (Almaani et al., 2017). However, the pathogenesis of LN remains unclear. As LN has complex clinical manifestations and no specific treatment, biomarkers and treatment targets are urgently needed. With the advancement of bioinformatics and metabolomics, increasing numbers of researchers are focusing on discovering biomarkers for the early diagnosis of LN. Due to their non-invasive nature, these methods may potentially serve as alternatives to renal biopsy. Beyond classical serum markers for LN, such as anti-double-stranded DNA (anti-dsDNA) antibodies and C1q, abnormal DNA methylation, non-coding RNA, and variations in levels of chemokines, interleukins, and urinary proteins may all serve as potential new biomarkers (Alduraibi and Tsokos, 2024). And the recent advent of gene testing and bioinformatics analysis has gradually elucidated associations between genes and diseases.

## 2 Materials and method

### 2.1 Data download

GSE32591 (Bethunaickan et al., 2011) and GSE113342 (Mejia-Vilet et al., 2019) are two sets of gene expression profile data for LN, downloaded from the official NCBI GEO website (https://www.ncbi.nlm.nih.gov/geo/) (Barrett et al., 2007) using the GEOquery package in R (Davis and Meltzer, 2007). GSE32591 and GSE113342 were divided into tubular interstitial (TUB) and glomerular (GLOM) gene expression groups. The gene expression profiles of patients with LN and patients in the control group are presented in Table 1.

**TABLE 1:** GEO database of LN patient data.

ID	GLOM (LN)	GLOM (LD)	TUB (LN)	*TUB* (LD)
GSE32591	32	14	32	15
GSE113342	28	6	28	10

TUB, tubular interstitial; GLOM, glomerular.

### 2.2 Co-differentially expressed genes (DEGs)

To investigate the effect of gene expression on patients from the LN and normal sample groups, data from GSE32591 and GSE113342 were divided into TUB and GLOM categories. The limma package in R was used to analyze the differences between groups (Ritchie et al., 2015). DEGs were determined using an adjusted *p*-value threshold of <0.05. Specifically, genes with logFC >1 and an adjusted *p*-value <0.05 were deemed upregulated DEGs, while those with logFC < −1 and an adjusted *p*-value <0.05 were categorized as downregulated DEGs. The DEGs identified across the four datasets were compared and intersected to identify the co-DEGs. These DEGs were visualized using the ggplot2 package in R (Maag, 2018). Additionally, the effects of shared DEGs on patient stratification were assessed using the heatmap package in R.

### 2.3 co-DEG function and pathway enrichment analysis

In extensive gene enrichment studies, gene ontology (GO) functional annotation analysis was used to explore biological processes (BP), molecular functions (MF), and cellular components (CC). The Kyoto encyclopedia of genes and genomes (KEGG) database (Kanehisa and Goto, 2000) served as a repository for information related to genomes, biological pathways, diseases, and drugs. The clusterProfiler package in R was used to perform GO function annotation and KEGG pathway enrichment analyses of the identified co-DEGs (Wu et al., 2021). A *p* < 0.05 was considered the threshold for statistical significance.

### 2.4 Protein-protein interaction (PPI) network construction

The STRING database, comprising established and predicted PPIs, was used to construct a PPI network (Szklarczyk et al., 2019). The PPI network model was visualized using Cytoscape software (Shannon et al., 2003). Local clusters within the network, characterized by closely connected interactions, could suggest molecular complexes associated with biological functions. Pearson’s correlation coefficients were calculated between the expression levels of IRF8 and other genes to analyze the gene expression profile data from the four datasets, with statistical significance set at *p* < 0.05. Genes demonstrating a significant correlation with IRF8 in at least three datasets were identified as IRF8-associated genes. Selecting IRF8-associated genes was depicted using the VennDiagram package in R (Chen and Boutros, 2011). DAVID, an online resource located at http://david.abcc.ncifcrf.gov/, integrates biological datasets and analytical tools to facilitate the construction of extensive gene or protein lists, provides detailed annotations of biological functions, and supports the analysis of biological information about these lists. In this study, DAVID was utilized for function annotation and pathway enrichment analysis, considering a *p* < 0.05 to be statistically significant.

### 2.5 Diagnostic prediction, model construction, and verification

Random forest is a bagging-based ensemble learning method used for regression, classification, and other applications (Brieuc et al., 2018). It is highly accurate, rapidly trained, and easy to implement, and it also performs variable-importance ranking. We intersected IRF8-related genes with co-DEGs to identify key genes and further evaluated the impact of their expression levels on patient diagnosis. The random forest algorithm was used for classification and was implemented using the RandomForest package in WEKA. Feature selection was performed, and a diagnosis prediction model was built using the RandomForest package in R (Svetnik et al., 2003), resulting in a classifier for the feature genes. During the construction and validation of the classifier, the training set comprised GLOM samples from the initial analysis of GSE32591, which included 32 LN and 14 normal tissues, along with TUB samples from the same dataset and GLOM and TUB samples from the GSE113342 dataset. The classifier model was then tested, and the pROC package in R was used to generate ROC curves and compute the AUC (Robin et al., 2011).

### 2.6 Immune infiltration analysis

CIBERSORT, available at https://cibersort.stanford.edu/, utilizes linear support vector regression to deconvolute immune cell subtype expression from gene expression matrices based on predefined reference profiles and a set of gene expression features representing 22 white blood cell subtypes (Newman et al., 2019). In this study, RNA-Seq data were used to estimate the levels of immune cell infiltration. The CIBERSORT algorithm was subsequently employed to evaluate the relationship between the co-DEGs and immune cell infiltration.

### 2.7 Mice

Female MRL/Mpj and MRL/lpr mice were obtained from Shanghai Jihui Laboratory Animal Care Co. Ltd (Shanghai, China) and housed in a pathogen-free facility at Fudan University. The Institutional Animal Care and Use Committee of Fudan University approved all animal experiments.

### 2.8 Quantitative real-time polymerase chain reaction (qRT-PCR)

RNA extraction from tissue samples was performed using TRIzol Reagent (15,596,026, Invitrogen, United States) following the manufacturer’s guidelines. The PrimeScript RT Reagent Kit (Takara, Japan) was used for cDNA synthesis. Expression levels of IRF8 were quantified using TB Green Premix Ex Taq II (Takara, Japan) on a QuantStudio 6 Flex Real-Time PCR System (ABI, United States). Data were analyzed using the delta-delta Ct method. Primer sequences are provided in Supplementary Table S1.

### 2.9 Western blot

Samples were lysed using RIPA lysis buffer (P0013C, Beyotime, China). The protein concentrations were measured using a BCA assay kit (P0010S, Beyotime, China). Proteins were then analyzed through standard Western blotting techniques. Membranes were incubated overnight at 4°C with primary antibodies, specifically anti-interferon regulatory factor 8 (IRF-8) (1:1,000, 5,628, CST, United States) and GAPDH (1:1,000, 2,118, CST, United States). Following washing, the membranes were incubated with goat anti-rabbit IgG secondary antibody (1:2000, 7,074, CST, United States) for 1 h at room temperature. Detection was carried out using ECL reagent (Millipore, United States), and images were obtained using a LAS-3000 imager (Fujifilm, Japan). Image quantification was conducted using Photoshop (Adobe).

### 2.10 Regulatory networks and target drugs of hub genes

The potential influence of drugs on the expression of hub genes was investigated using the drug–gene interaction database (DGIdb), which aggregates drug-gene interaction information from 30 different sources (Cotto et al., 2018). Additionally, the Cytoscape software was employed to facilitate a more detailed analysis of the drug network, enhancing the examination of the interactions within (Shannon et al., 2003).

### 2.11 Statistical analysis

Data processing and analysis were conducted using the R software (version 4.0.2; R Core Team, Vienna, Austria). Graphical representations were generated using the ggplot2 package. The pROC package in R (Robin et al., 2011) was used to create ROC curves, compute the AUC, assess the accuracy of risk scores, and predict prognosis. Statistical comparisons between the two groups were conducted using the Student's t-test, with a *p* < 0.05 considered statistically significant.

## 3 Results

### 3.1 Co-DEGs

**FIGURE 1:**
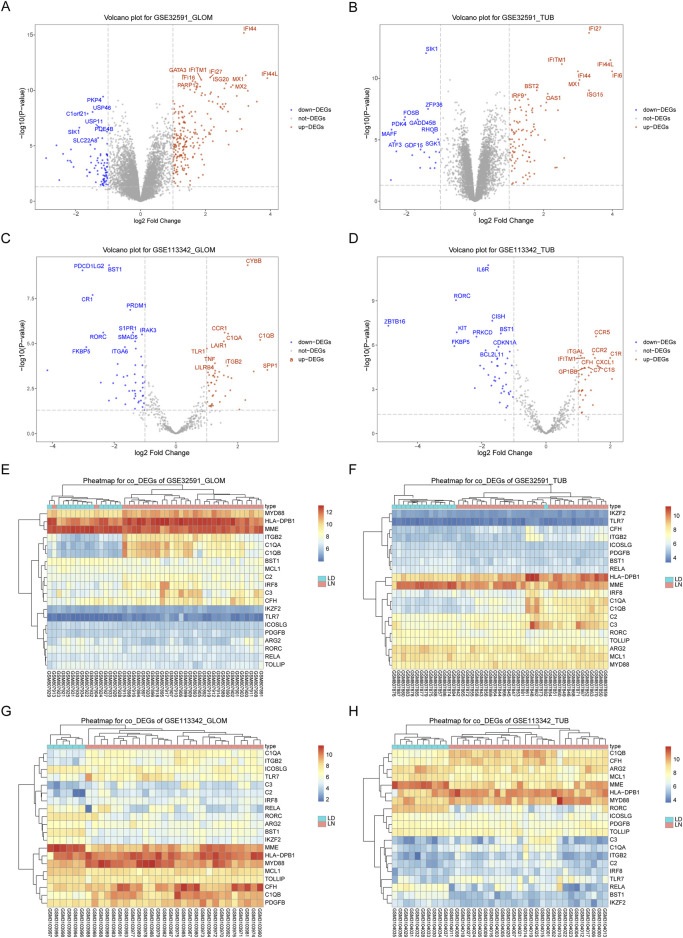
Differentially Expressed Genes in GSE32591 and GSE113342. **(A–D)** Volcano plots showing differentially expressed genes with log2(Fold Change) on the x-axis and -log10(p.adjust) on the y-axis. **(E–H)** Heatmaps showing expression levels of differentially expressed genes across patient samples.

**FIGURE 2:**
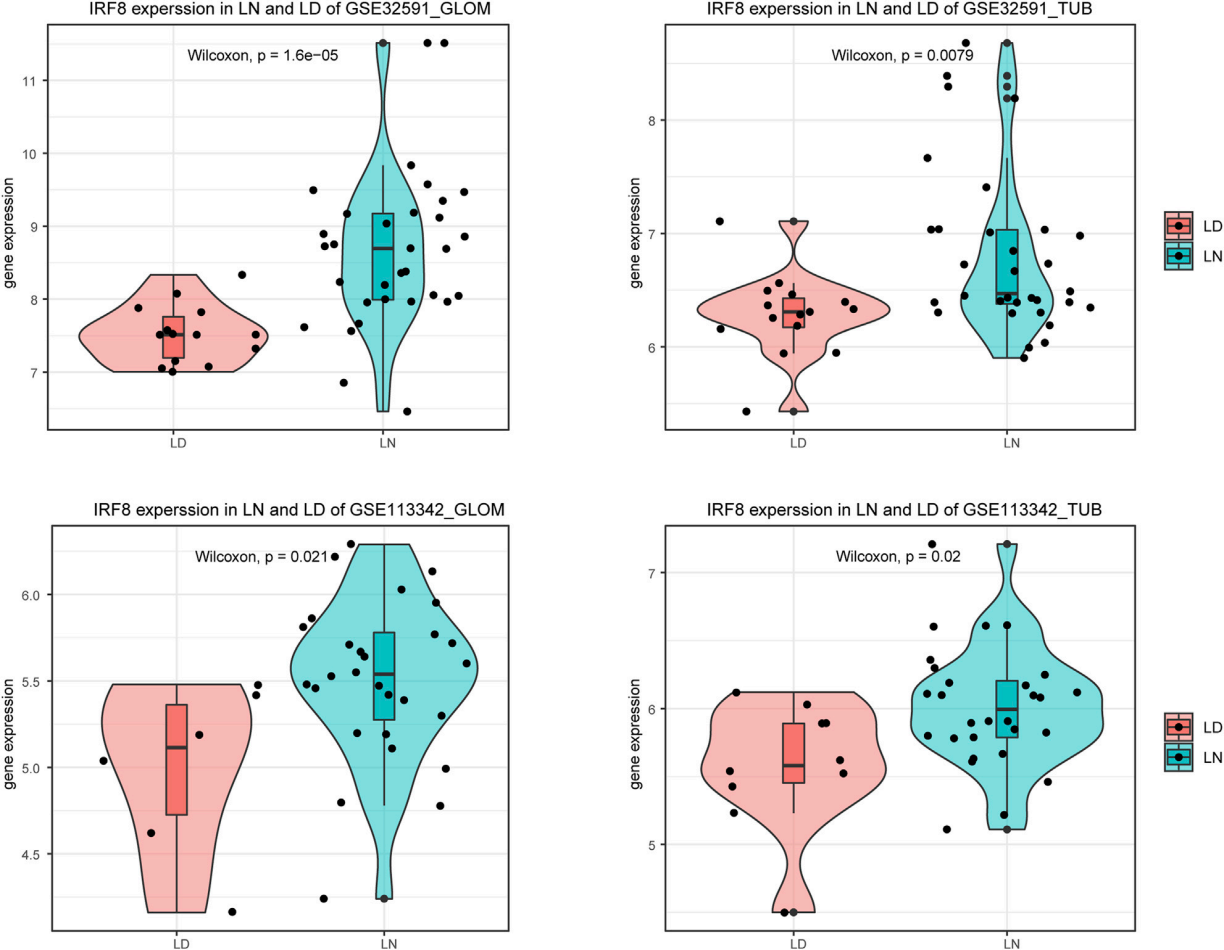
Expression levels of *IRF8* across datasets.

We used the limma package to perform differential expression analysis to investigate the impact of gene expression in patients with LN compared to normal controls. This allowed us to identify DEGs, categorized into upregulated and downregulated DEGs across four sample groups, as demonstrated in Figures 1A–D and Supplementary Table S2. We then intersected the downregulated DEGs from dataset GSE32591 with those from GSE113342 and the upregulated DEGs from GSE32591 with those from GSE113342. This process identified 20 co-DEGs. To assess the relevance of these co-DEGs for clinical diagnosis, classification heatmaps were generated (Figures 1E–H). The heatmaps demonstrated that the 20 co-DEGs differentiated the disease samples from the normal ones. Furthermore, statistical analysis of the gene expression levels within the co-DEGs indicated a significant elevation in IRF8 expression in the LN group (Figure 2).

### 3.2 Functional enrichment analysis of co-DEGs

**FIGURE 3:**
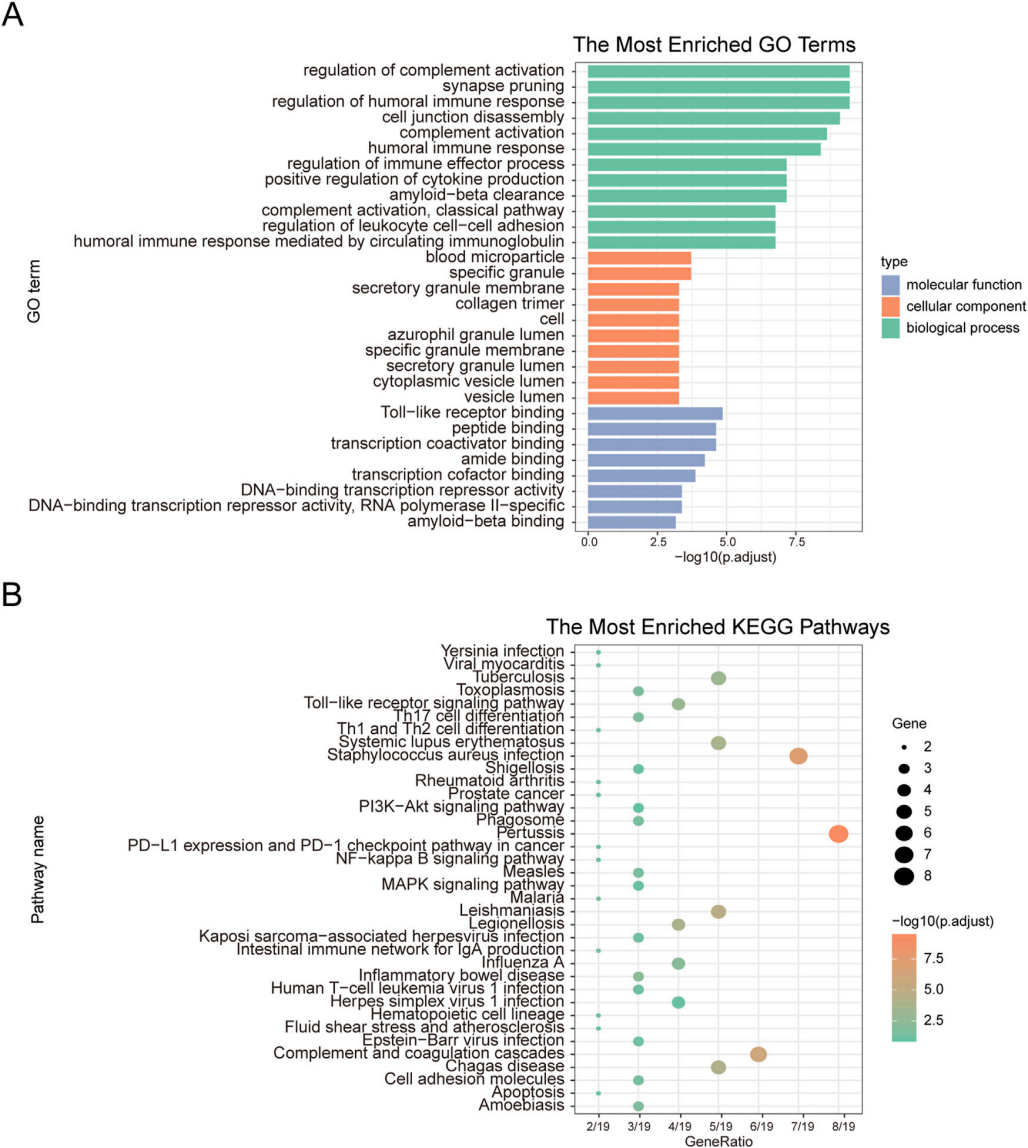
GO functional enrichment analysis and KEGG pathway enrichment analysis. **(A)** GO functional enrichment analysis. The x-axis represents -log(p.adjust), and the y-axis represents GO terms. **(B)** KEGG pathway enrichment analysis. The x-axis represents generation, and the y-axis represents pathway names. The size of the nodes indicates the number of genes enriched in the pathway, while the node color represents -log10(*p*-value).

To explore the relationship between co-DEGs and various BP, MF, CC, and pathways, we performed functional enrichment analysis of the 20 identified co-DEGs. These genes were primarily associated with BP, such as the regulation of complement activation, synapse pruning, regulation of the humoral immune response, and cell junction disassembly. Regarding MF, the co-DEGs were enriched in activities such as toll-like receptor binding, peptide binding, transcription coactivator binding, and amide binding. Furthermore, they were linked to CC, including blood microparticles, specific granules, secretory granule membranes, and collagen trimers (Figure 3A). The co-DEGs were also enriched in biological pathways, such as those involved in pertussis, *Staphylococcus aureus* infection, complement and coagulation cascades, Leishmaniasis, and Chagas disease (Figure 3B).

### 3.3 Construction of PPI network and module extraction

**FIGURE 4:**
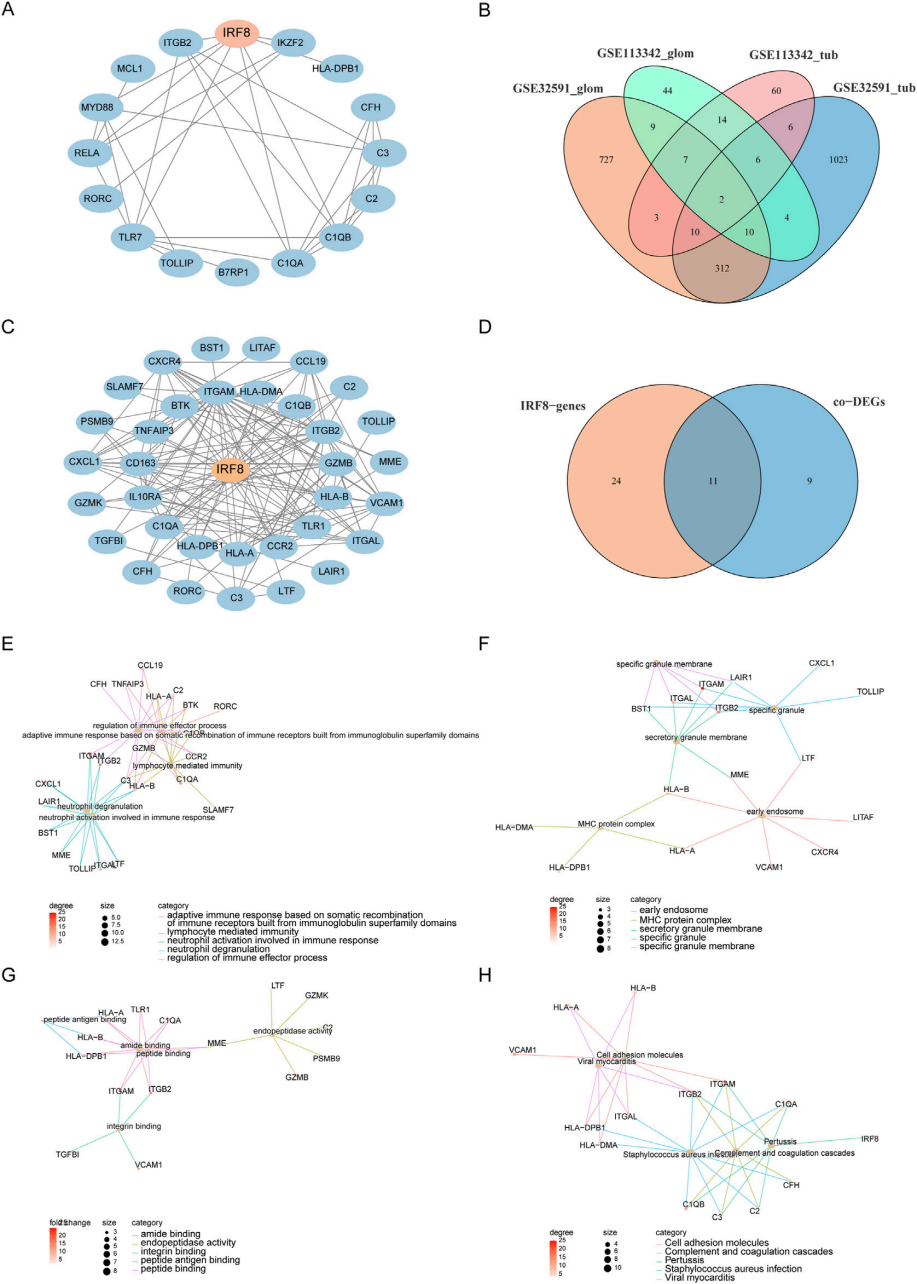
PPI and Functional Analysis. **(A)** PPI network of co-DEGs. **(B)** IRF8-related genes obtained from four datasets. **(C)** PPI network of IRF8-related genes. **(D)** Intersection of IRF8-related genes and co-DEGs. **(E–H)** Functional annotation and pathway enrichment analysis of genes in functional modules using DAVID, with node color representing the degree of IRF8-related gene nodes.

We used the STRING database to construct a PPI network (Figure 4A) consisting of 16 genes and 34 interaction pairs to explore the interactions between co-DEGs. Within this network, the IRF8 node exhibited the highest degree of connectivity. To explore the impact of IRF8 on LN, we calculated genes highly correlated with IRF8 expression levels and identified 1080 IRF8-related genes in GSE32591-GLOM, 1,373 in GSE32591-TUB, 96 in GSE113342-GLOM, and 108 in GSE113342-TUB. An intersection of the four IRF8-related gene sets revealed 35 genes significantly correlated with IRF8 across at least three datasets (Figure 4B). We extracted a PPI network for these 35 IRF8-related genes using the STRING database (Figure 4C), which included 35 genes and 139 interactions, encompassing 11 co-DEGs (Figure 4D). C1QA and C1QB, validated by the literature to be related to LN (Wu et al., 2020), were among these. This indicates a close correlation between IRF8-related genes and LN. Subsequent analysis of the biological functions affected by IRF8-related genes revealed that these 35 genes were mainly enriched in BP, such as regulation of immune effector processes, adaptive immune responses based on somatic recombination of immune receptors built from immunoglobulin superfamily domains, lymphocyte-mediated immunity, and neutrophil degranulation (Figure 4E). They were also enriched in CC-like specific granules, MHC protein complexes, specific granule membranes, and secretory granule membranes (Figure 4F) and MF, including peptide, amide, peptide antigen, and integrin binding (Figure 4G). Furthermore, they affected biological pathways, such as *Staphylococcus aureus* infection, pertussis, complement and coagulation cascades, and cell adhesion molecules (Figure 4H).

### 3.4 Characteristic gene screening and diagnostic value assessment

**FIGURE 5:**
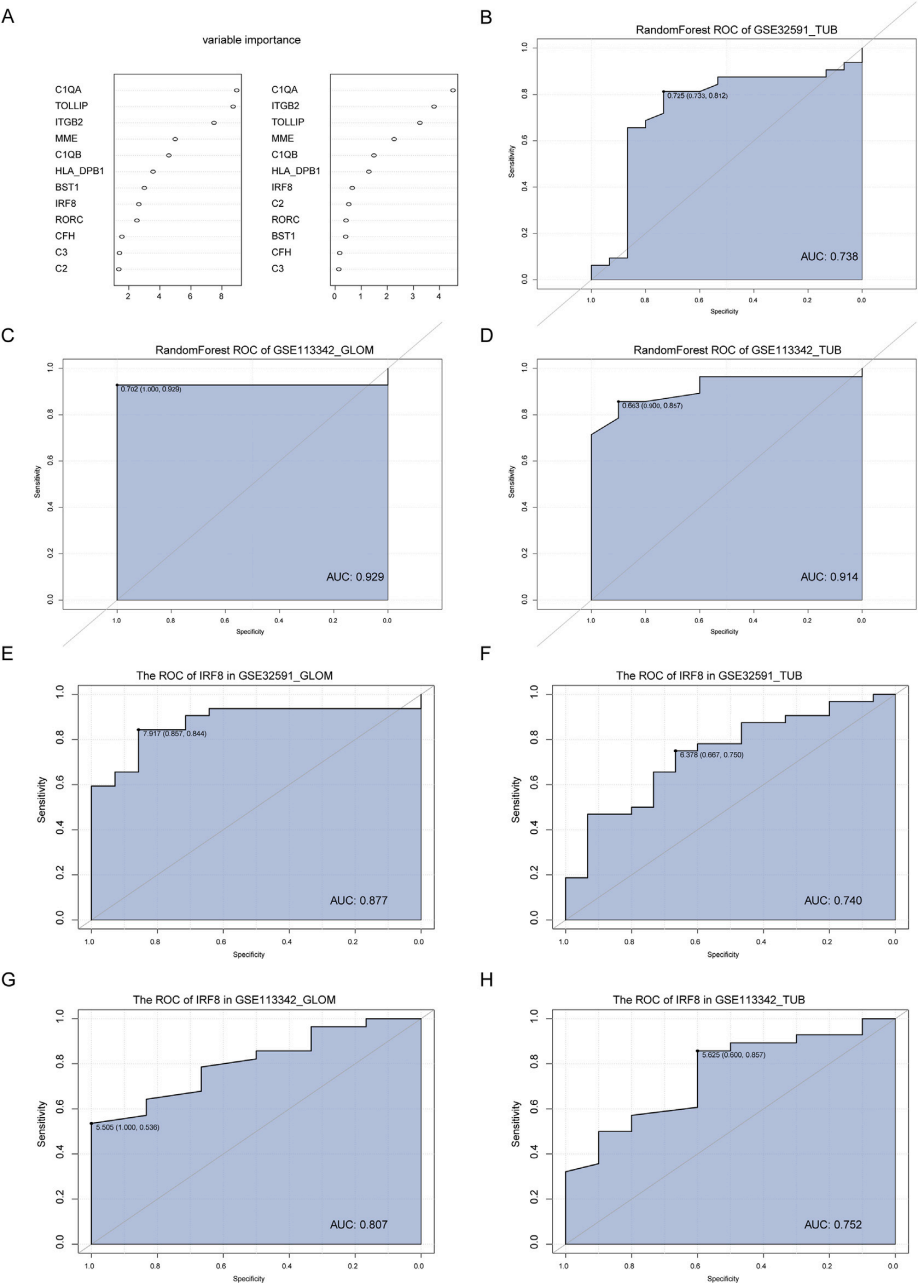
Construction and Validation of the Diagnostic Classifier. **(A)** Feature selection and diagnostic classifier model construction using the random forest algorithm on the GSE32591-GLOM dataset, ranking the importance scores of 11 key feature genes. **(B–D)** Testing the diagnostic classifier model on three additional datasets: GSE32591_TUB, GSE113342_GLOM, and GSE113342_TUB, with ROC curves plotted. **(E–H)** ROC curves of the IRF8 gene in GSE32591_GLOM, GSE32591_TUB, GSE113342_GLOM, and GSE113342_TUB datasets.

By intersecting IRF8-related genes with the co-DEGs, we identified 11 key genes. To assess their diagnostic value for LN, we used the RandomForest feature selection method within WEKA on the GSE32591-GLOM dataset and constructed a diagnostic classifier using the RandomForest package in R, resulting in an importance score for the 11 feature genes (Figure 5A). We validated the diagnostic classifier using three datasets: GSE32591-TUB, GSE113342-GLOM, and GSE113342-TUB and plotted ROC curves. The results illustrated that the AUC for GSE32591-TUB was 0.738 (Figure 5B), for GSE113342-GLOM was 0.929 (Figure 5C), and for GSE113342-TUB was 0.914 (Figure 5D), indicating that our diagnostic classifier can effectively differentiate disease samples from normal samples. Furthermore, we analyzed whether IRF8 could distinguish diseased samples from normal samples. The results indicated that the AUC for IRF8 in GSE32591-GLOM was 0.877 (Figure 5E), in GSE32591-TUB was 0.744 (Figure 5F), in GSE113342-GLOM was 0.807 (Figure 5G), and in GSE113342-TUB was 0.752 (Figure 5H), respectively, demonstrating that the expression level of IRF8 significantly affected LN diagnosis.

### 3.5 CIBERSORT immune infiltration analysis

**FIGURE 6:**
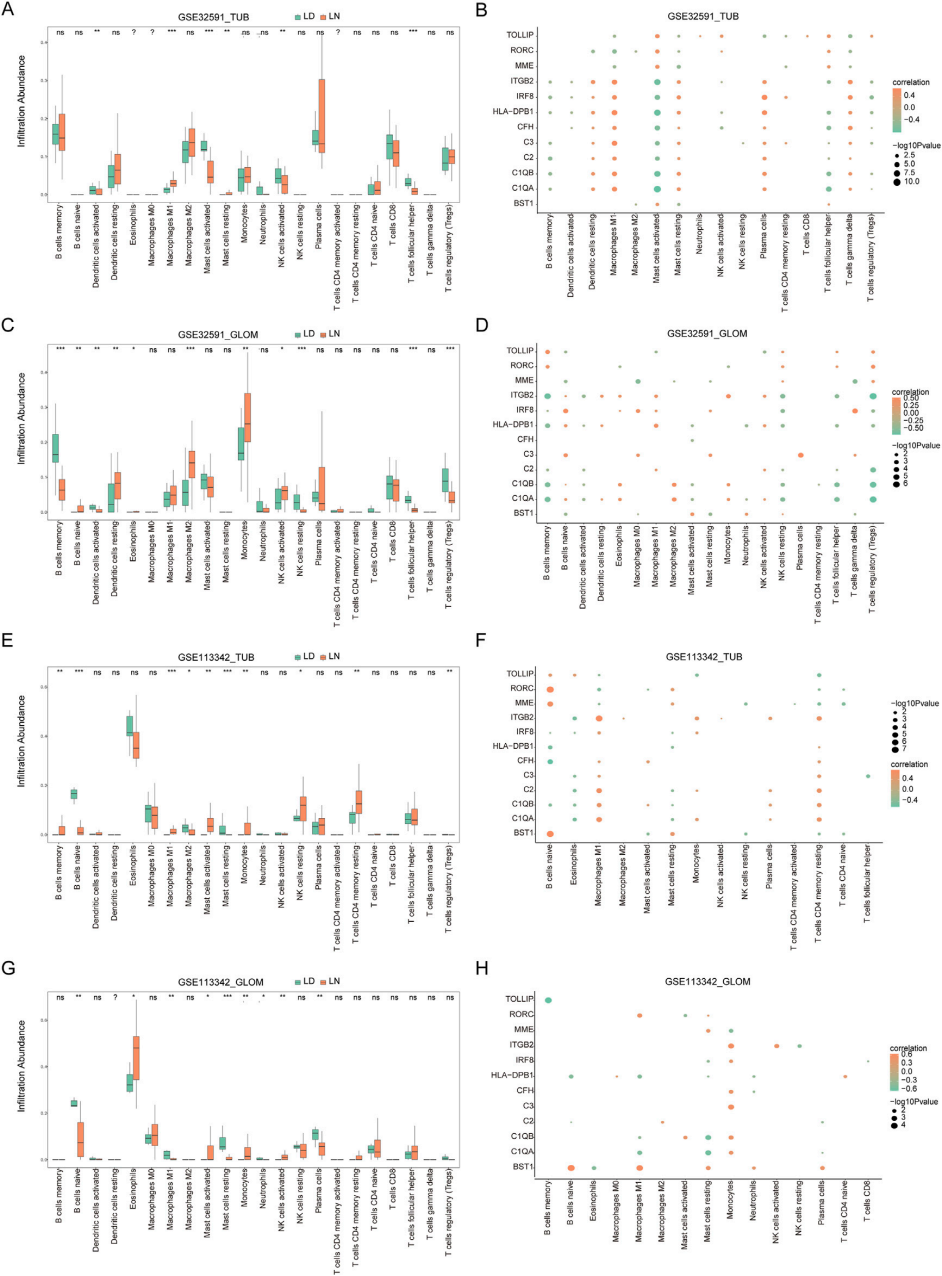
Immune Infiltration Analysis and Correlation between Key Genes and Immune Cells. **(A)** Immune infiltration in the GSE32591_TUB group, with the x-axis representing immune cells and the y-axis representing immune cell abundance. Asterisks indicate significance levels: *p < 0.05, **p < 0.01, ***p < 0.001. Similar plots are shown for GSE32591_GLOM **(C)**, GSE113342_TUB **(E)**, and GSE113342_GLOM **(G)**. **(B)** Correlation between 11 key feature genes and immune cells in the GSE32591_TUB group. The x-axis represents immune cells, and the y-axis represents feature genes, with orange indicating positive correlation and green indicating negative correlation. Node size represents the level of significance. Similar plots are shown for GSE32591_GLOM **(D)**, GSE113342_TUB **(F)**, and GSE113342_GLOM **(H)**.

The CIBERSORT algorithm was utilized to evaluate immune cell infiltration differences across two distinct RNA modification patterns. The analysis revealed substantial variations in immune cell populations between LN and LD groups. Specifically, in GSE32591-TUB, there were significant differences in the proportions of activated dendritic cells, M1 macrophages, activated and resting mast cells, activated NK cells, and follicular helper T cells (*p* < 0.05, Figure 6A). Moreover, a significant correlation was observed between key genes and the number of M1 macrophages and activated and resting mast cells (Figure 6B). In GSE32591-GLOM, memory B cells, naïve B cells, activated and resting dendritic cells, and eosinophils were significantly different (*p* < 0.05, Figure 6C), with memory and naïve B cells exhibiting significant correlations with key gene expression levels (Figure 6D). For GSE113342-TUB, significant differences were observed in the content of M1 and M2 macrophages, activated and resting mast cells, and monocytes (*p* < 0.05, Figure 6E); M1 macrophages and resting mast cells also demonstrated significant correlations with key gene expression levels (Figure 6F). Last, in GSE113342-GLOM, notable differences were detected in the levels of activated mast cells, resting mast cells, monocytes, neutrophils, and activated NK cells (*p* < 0.05, Figure 6G), with significant correlations between resting mast cells, monocytes, and multiple key genes.

### 3.6 Validation of IRF8 and levels of its related genes

**FIGURE 7:**
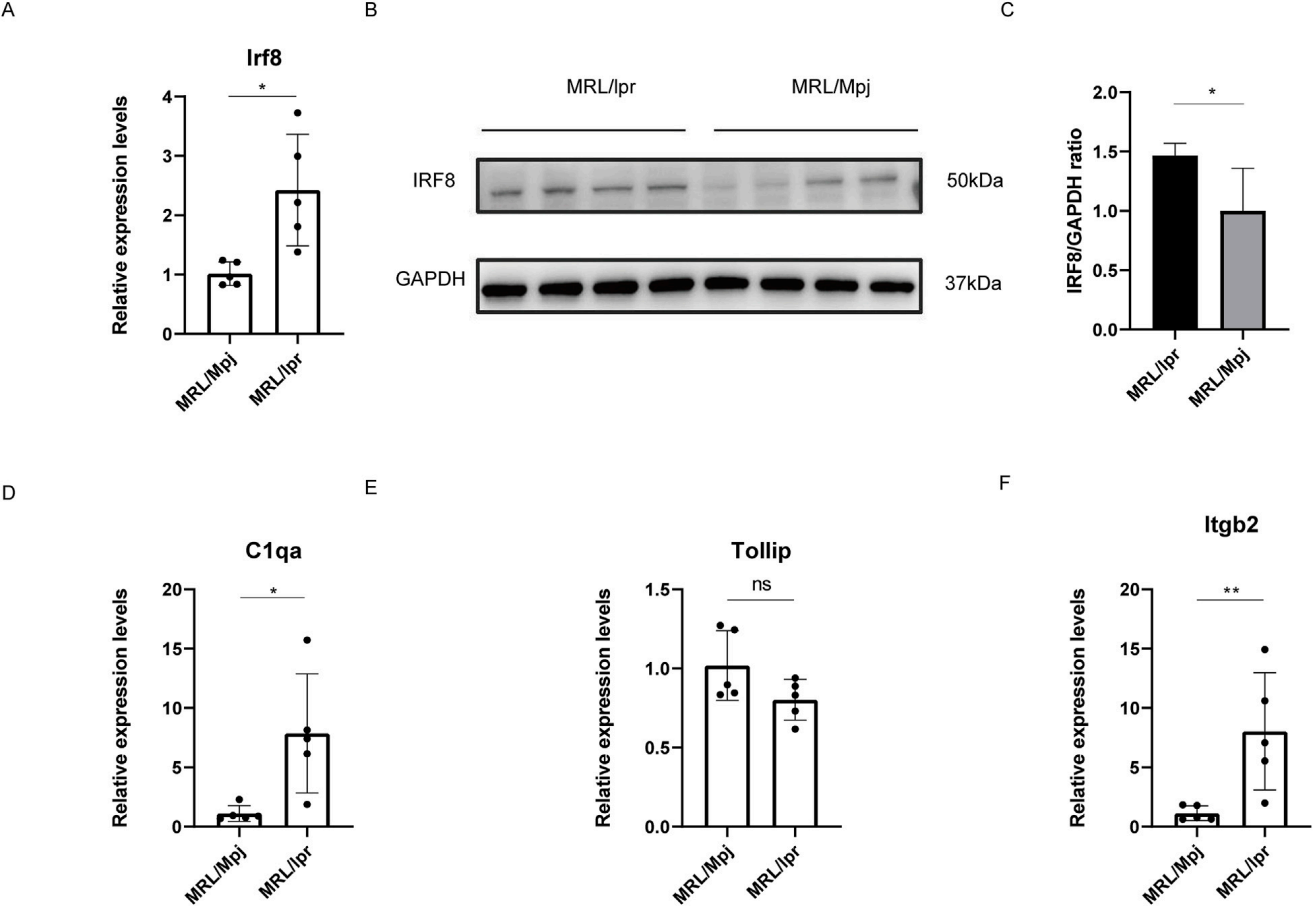
Validation of IRF8 Expression and Its Related Genes. **(A)** qRT-PCR validation for Irf8 (n = 5). **(B)** Western blot validation for IRF8 expression (n = 4). **(C)** The quantification of the Western blot bands of IRF8. **(D)** qRT-PCR validation for C1qa (n = 5). **(E)** qRT-PCR validation for Tollip (n = 5). **(F)** qRT-PCR validation for Itgb2 (n = 5). Significance levels are denoted as follows: *p < 0.05, **p < 0.01, ****p < 0.0001, with ‘ns’ indicating no significant difference.

Recent study have demonstrated that 18-week-old MRL/lpr mice displayed glomerular swelling and significant kidney inflammatory cell infiltration (Chen et al., 2023). Consequently, we assessed IRF8 expression levels in the kidneys of these mice using qRT-PCR and Western blot analysis (Figures 7A–C). The results showed that both gene and protein expression levels of IRF8 were significantly elevated in the kidneys of 18-week-old MRL/lpr mice compared to control mice. Additionally, we used qRT-PCR to evaluate the expression of C1qa, Tollip, and Itgb2 (Figures 7D–F). Our findings revealed that C1qa and Itgb2 expression levels were significantly upregulated, while Tollip showed no significant difference in the kidneys of 18-week-old MRL/lpr mice compared to control mice.

### 3.7 Analysis of drug regulatory network in LN

**FIGURE 8:**
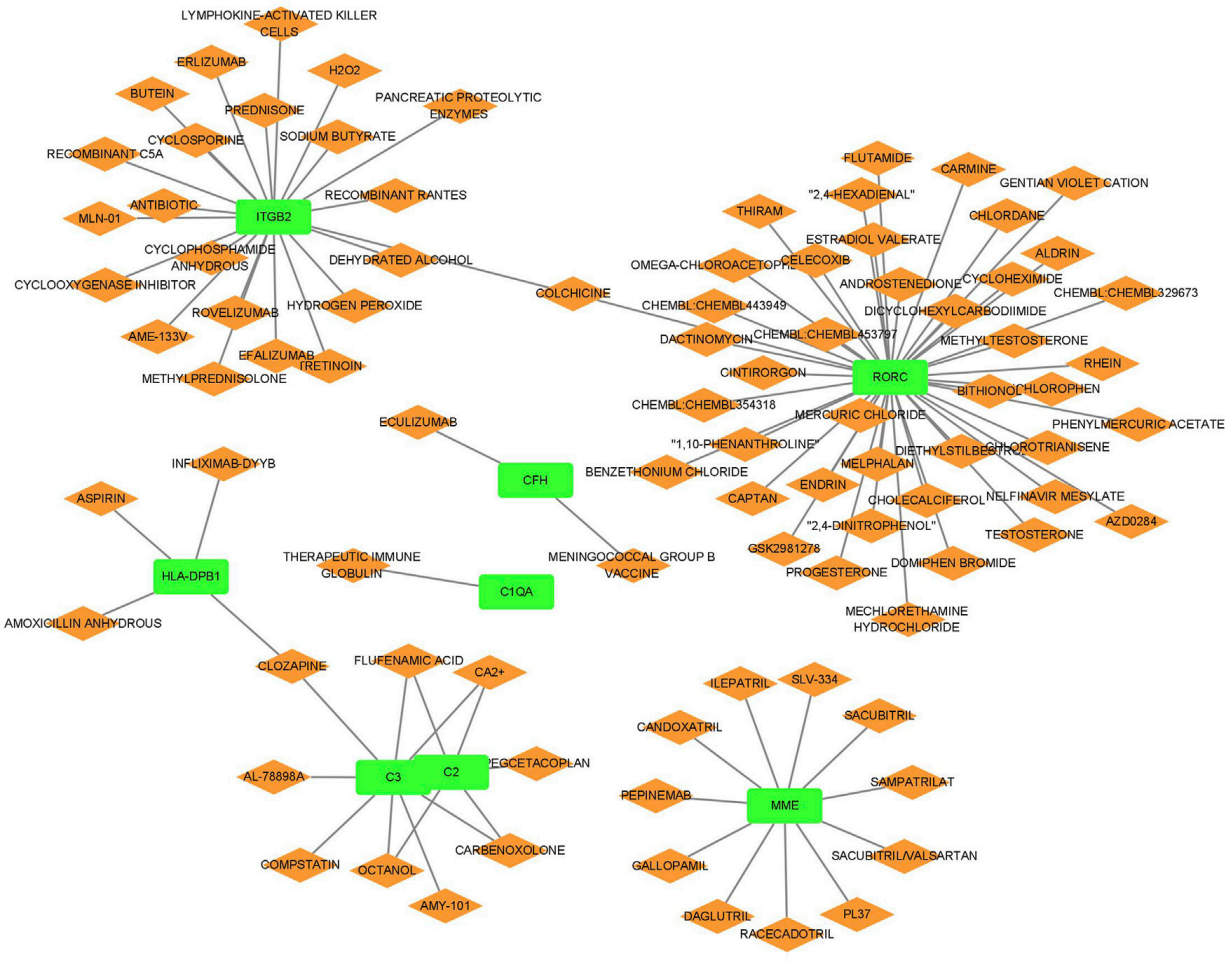
Drugs influencing hub gene expression or function.

The relationship between the biomarkers and drugs is presented in Figure 8. We identified several drugs targeting multiple genes integral to the disease pathway. Calcium ions (Ca2+), carbenoxolone, flufenamic acid, and octanol interacted with genes C2 and C3, suggesting a mechanism by which these drugs may influence the complement system, essential for immune responses. Similarly, clozapine targeted HLA-DPB1 and C3, potentially indicating its role in modulating immune functions and inflammatory responses. Furthermore, colchicine interacted with ITGB2 and RORC, underscoring its potential to regulate cellular adhesion processes and immune cell differentiation pathways. These insights suggest that these drugs may play a significant role in comprehensive strategies to treat conditions involving these critical pathways.

## 4 Discussion

LN affects ≤40% of all adults and ≤80% of all children with SLE and causes irreversible kidney damage. However, its pathogenesis is unclear, and no specific or sensitive biomarkers exist for its diagnosis or treatment. In clinical trials, only 30%–50% of patients enter remission, and 10%–20% develop ESRD within 10 years of diagnosis (Maria and Davidson, 2020). Therefore, it is vital to understand the pathology and molecular mechanisms underlying LN for its effective diagnosis and treatment. Microarray and bioinformatics analyses can clarify the molecular mechanisms underlying disease occurrence and development. In our study, by intersecting the DEGs across the datasets, we identified 20 co-DEGs. Further intersecting IRF8-related genes with these co-DEGs led us to identify 11 key genes. IRF8 was significantly upregulated in both GLOM and TUB groups. As a key transcription factor, IRF8 is vital for innate and adaptive immunity and contributes to cytokine production, particularly in the type I interferon pathway. These cytokines may lead to aberrant immune cell activation, resulting in the chronic inflammation commonly observed in SLE (Salloum and Niewold, 2011). Moreover, variations in the IRF8 gene increase susceptibility to SLE by regulating immune responses to environmental triggers (Cunninghame Graham et al., 2011; Lin et al., 2015; Sheng et al., 2015; Cai et al., 2017). This gene association highlights the potential of IRF8 as a biomarker for assessing SLE risk and disease progression. However, despite substantial research into the role of IRF8 in SLE, few studies have explored its involvement in LN, and its function in LN remains unclear. The complement system also plays a crucial role in SLE pathogenesis, particularly components such as C1QA, C1QB, C2, and C3, which are essential for clearing apoptotic debris and immune complexes, thereby reducing autoimmunity and systemic inflammation (Mitchell et al., 2002; Sun-Tan et al., 2010; Carlucci et al., 2016). Genetic polymorphisms in C1QA and C1QB have been associated with increased SLE susceptibility, affecting serum C1q levels and disease severity. The rs631090 SNP in the C1QB gene is linked to SLE, leading to lower C1q levels, which may result in inefficient clearance of immune complexes and apoptotic cells (Martens et al., 2009). C2 and C3 are key complement system components and are integral to the classical and alternative pathways. C2 deficiency impairs immune complex clearance and increases SLE risk (Lundtoft et al., 2022). Serum C3 levels are significantly heritable and identifies specific genetic variants within the C3 gene associated with both serum levels and SLE susceptibility (Rhodes et al., 2009). In our analysis, the enrichment of the complement and coagulation cascade pathways further supports the critical role of the complement system in SLE. Complement factor H (CFH) primarily regulates the alternative complement pathway and prevents uncontrolled complement activation and tissue damage. Studies have demonstrated that CFH deficiency models exhibit severe disease progression, with increased proteinuria, elevated BUN levels, and significant kidney damage due to uncontrolled complement activity and immune complex deposition. Despite their crucial role, cross-population genetic studies suggest that individual genetic variations may not significantly affect disease susceptibility or progression (Bao et al., 2011; Li Q.-Y. et al., 2023; Ma et al., 2023). Retinoic acid-related orphan receptor C (RORC) primarily functions in Th17 cell differentiation and may contribute to SLE pathogenesis by regulating interleukin (IL)-17 production. RORC expression is lower in patients with SLE compared to healthy individuals, suggesting an imbalance in immune regulation, particularly in the interaction between IL-23 and STAT3, which may influence clinical symptoms and treatment outcomes in SLE (El-Karaksy et al., 2016; Kluger et al., 2017). The ITGB2 gene encodes the β2 integrin subunit, a key component of the β2 integrin family that promotes cell adhesion and immune responses (Li H. et al., 2023). Membrane metalloendopeptidase, also known as neprilysin, is involved in various physiological and pathological processes, including cancer and autoimmune diseases (Ding et al., 2023). HLA-DPB1 alleles are associated with SLE and specific autoantibodies. Some studies have found that certain HLA-DPB1 alleles are related to anticardiolipin and anti-β2 glycoprotein I antibodies, suggesting their involvement in autoimmune responses. Additionally, these alleles are associated with specific clinical features of SLE, such as livedo reticularis and Raynaud’s phenomenon, further contributing to the clinical diversity of the disease (Korioth et al., 1992; Sebastiani et al., 2003). To date, no studies have investigated the relationship between BST1 and TOLLIP genes and SLE.

By integrating IRF8-related genes with the co-DEGs, we identified 11 key genes and assessed their potential as diagnostic markers for LN using the random forest algorithm in WEKA, based on the GSE32591-GLOM dataset. We tested the diagnostic classifier on three independent datasets with AUC values of 0.738, 0.929, and 0.914 for GSE32591-TUB, GSE113342-GLOM, and GSE113342-TUB, respectively. These results indicate that this method can effectively distinguish the disease. Additionally, the diagnostic significance of IRF8 was confirmed, with AUC values ranging from 0.744 to 0.877 across the different datasets.

We further explored the effect of co-DEGs on immune infiltration in LN. The analysis of immune cell variations between the LN and control groups revealed significant differences. B cells are integral to the pathogenesis of SLE, primarily through autoantibody production, antigen presentation, and immune response modulation. Abnormalities in B-cell tolerance, signaling, and cytokine production contribute to atypical B-cell activation and differentiation, advancing disease progression (Yap and Chan, 2019). Macrophages and dendritic cells are essential for SLE, specifically in the LN. Macrophages originate from monocytes and are crucial for phagocytosis, tissue remodeling, and cytokine production. In LN, renal macrophages, particularly the resident F4/80^hi^ population, proliferate and assume an inflammatory phenotype that causes tissue damage and fibrosis. Efficient antigen-presenting dendritic cells infiltrate the kidneys and form tertiary lymphoid structures, exacerbating the local inflammation. The disrupted functions of these cell types in SLE heighten immune responses and impede resolution; therefore, they are identified as primary targets for therapeutic strategies to preserve renal function and mitigate disease progression (Maria and Davidson, 2017).

This study, primarily focused on validating the differential expression of the IRF8 gene in lupus mouse models, encounters several limitations. It does not include validation in human tissues nor does it explore the specific roles of the IRF8 gene in LN. Additionally, the tissue sample size is insufficient, requiring enlargement to more robustly confirm and generalize the findings. Lastly, the conclusions of the study are based exclusively on a single type of omics analysis, which might overlook essential biological interactions and pathways that could be uncovered through a comprehensive multi-omics approach.

## 5 Conclusion

In conclusion, this study has successfully identified IRF8 and IRF8-related genes that possess significant diagnostic value for LN. This research provides novel insights into the diagnosis and treatment of LN and lays a solid foundation for future empirical investigations.

## Data Availability

The datasets presented in this study can be found in online repositories. The names of the repository/repositories and accession number(s) can be found in the article/Supplementary Material.

## References

[B1] Alduraibi F. K. Tsokos G. C. (2024). Lupus nephritis biomarkers: a critical review. IJMS 25, 805. 10.3390/ijms25020805 38255879 PMC10815779

[B2] Almaani S. Meara A. Rovin B. H. (2017). Update on lupus nephritis. Clin. J. Am. Soc. Nephrol. 12, 825–835. 10.2215/CJN.05780616 27821390 PMC5477208

[B3] Bao L. Haas M. Quigg R. J. (2011). Complement factor H deficiency accelerates development of lupus nephritis. J. Am. Soc. Nephrol. 22, 285–295. 10.1681/ASN.2010060647 21148254 PMC3029901

[B4] Barrett T. Troup D. B. Wilhite S. E. Ledoux P. Rudnev D. Evangelista C. (2007). NCBI GEO: mining tens of millions of expression profiles--database and tools update. Nucleic Acids Res. 35, D760–D765. 10.1093/nar/gkl887 17099226 PMC1669752

[B5] Bethunaickan R. Berthier C. C. Ramanujam M. Sahu R. Zhang W. Sun Y. (2011). A unique hybrid renal mononuclear phagocyte activation phenotype in murine systemic lupus erythematosus nephritis. J. Immunol. 186, 4994–5003. 10.4049/jimmunol.1003010 21411733 PMC3159403

[B6] Brieuc M. S. O. Waters C. D. Drinan D. P. Naish K. A. (2018). A practical introduction to Random Forest for genetic association studies in ecology and evolution. Mol. Ecol. Resour. 18, 755–766. 10.1111/1755-0998.12773 29504715

[B7] Cai X. Huang W. Liu X. Wang L. Jiang Y. (2017). Association of novel polymorphisms in TMEM39A gene with systemic lupus erythematosus in a Chinese Han population. BMC Med. Genet. 18, 43. 10.1186/s12881-017-0405-8 28427360 PMC5399404

[B8] Carlucci F. Ishaque A. Ling G. S. Szajna M. Sandison A. Donatien P. (2016). C1q modulates the response to TLR7 stimulation by pristane-primed macrophages: implications for pristane-induced lupus. J. Immunol. 196, 1488–1494. 10.4049/jimmunol.1401009 26773156 PMC4745139

[B9] Chen H. Boutros P. C. (2011). VennDiagram: a package for the generation of highly-customizable Venn and Euler diagrams in R. BMC Bioinforma. 12, 35. 10.1186/1471-2105-12-35 PMC3041657 21269502

[B10] Chen Q. Xiang M. Gao Z. Lvu F. Sun Z. Wang Y. (2023). The role of B-cell ferroptosis in the pathogenesis of systemic lupus erythematosus. Clin. Immunol. 256, 109778. 10.1016/j.clim.2023.109778 37730009

[B11] Cotto K. C. Wagner A. H. Feng Y.-Y. Kiwala S. Coffman A. C. Spies G. (2018). DGIdb 3.0: a redesign and expansion of the drug–gene interaction database. Nucleic Acids Res. 46, D1068–D1073. 10.1093/nar/gkx1143 29156001 PMC5888642

[B12] Cunninghame Graham D. S. Morris D. L. Bhangale T. R. Criswell L. A. Syvänen A.-C. Rönnblom L. (2011). Association of NCF2, IKZF1, IRF8, IFIH1, and TYK2 with systemic lupus erythematosus. PLoS Genet. 7, e1002341. 10.1371/journal.pgen.1002341 22046141 PMC3203198

[B13] Davis S. Meltzer P. S. (2007). GEOquery: a bridge between the gene expression Omnibus (GEO) and BioConductor. Bioinformatics 23, 1846–1847. 10.1093/bioinformatics/btm254 17496320

[B14] Ding J. Li C. Shu K. Chen W. Cai C. Zhang X. (2023). Membrane metalloendopeptidase (MME) is positively correlated with systemic lupus erythematosus and may inhibit the occurrence of breast cancer. PLoS ONE 18, e0289960. 10.1371/journal.pone.0289960 37585411 PMC10431625

[B15] El-Karaksy S. M. Raafat H. A. Abadir M. N. Y. Hanna M. O. F. (2016). Down-regulation of expression of retinoid acid-related orphan receptor C (RORC) in systemic lupus erythematosus. J. Recept. Signal Transduct. 36, 207–212. 10.3109/10799893.2015.1075042 26498317

[B16] Kanehisa M. Goto S. (2000). KEGG: kyoto encyclopedia of genes and genomes. Nucleic Acids Res. 28, 27–30. 10.1093/nar/28.1.27 10592173 PMC102409

[B17] Kluger M. A. Nosko A. Ramcke T. Goerke B. Meyer M. C. Wegscheid C. (2017). RORγt expression in Tregs promotes systemic lupus erythematosus via IL-17 secretion, alteration of Treg phenotype and suppression of Th2 responses. Clin. Exp. Immunol. 188, 63–78. 10.1111/cei.12905 27880975 PMC5343349

[B18] Kong J. Li L. Zhimin L. Yan J. Ji D. Chen Y. (2019). Potential protein biomarkers for systemic lupus erythematosus determined by bioinformatics analysis. Comput. Biol. Chem. 83, 107135. 10.1016/j.compbiolchem.2019.107135 31751880

[B19] Korioth F. Hartung K. Deicher H. Frey J. (1992). A new HLA-DPB1 allele from a patient with systemic lupus erythematosus. Tissue Antigens 39, 216–219. 10.1111/j.1399-0039.1992.tb01938.x 1529429

[B20] Li H. Zhang X. Shang J. Feng X. Yu L. Fan J. (2023a). Identification of NETs-related biomarkers and molecular clusters in systemic lupus erythematosus. Front. Immunol. 14, 1150828. 10.3389/fimmu.2023.1150828 37143669 PMC10151561

[B21] Li Q.-Y. Lv J.-M. Liu X.-L. Li H.-Y. Yu F. (2023b). Association of C-reactive protein and complement factor H gene polymorphisms with risk of lupus nephritis in Chinese population. World J. Clin. Cases 11, 2934–2944. 10.12998/wjcc.v11.i13.2934 37215422 PMC10198093

[B22] Lin J. Wang Y. Liu C. Lin Y. Lin J. Lin Y. (2015). Association of IRF 8 gene polymorphisms with autoimmune thyroid disease. Eur. J. Clin. Investig. 45, 711–719. 10.1111/eci.12463 25989711

[B23] Lundtoft C. Sjöwall C. Rantapää‐Dahlqvist S. Bengtsson A. A. Jönsen A. Pucholt P. (2022). Strong association of combined genetic deficiencies in the classical complement pathway with risk of systemic lupus erythematosus and primary sjögren’s syndrome. Arthritis and Rheumatology 74, 1842–1850. 10.1002/art.42270 35729719 PMC9828039

[B24] Ma Z. Mao C. Jia Y. Yu F. Xu P. Tan Y. (2023). ADAMTS7-Mediated complement factor H degradation potentiates complement activation to contributing to renal injuries. Clin. J. Am. Soc. Nephrol. 34, 291–308. 10.1681/ASN.0000000000000004 PMC10103097 36735376

[B25] Maag J. L. V. (2018). gganatogram: an R package for modular visualisation of anatograms and tissues based on ggplot2. F1000Res 7, 1576. 10.12688/f1000research.16409.2 30467523 PMC6208569

[B26] Maria N. I. Davidson A. (2017). Renal macrophages and dendritic cells in SLE nephritis. Curr. Rheumatol. Rep. 19, 81. 10.1007/s11926-017-0708-y 29119288

[B27] Maria N. I. Davidson A. (2020). Protecting the kidney in systemic lupus erythematosus: from diagnosis to therapy. Nat. Rev. Rheumatol. 16, 255–267. 10.1038/s41584-020-0401-9 32203285

[B28] Martens H. A. Zuurman M. W. De Lange A. H. M. Nolte I. M. Van Der Steege G. Navis G. J. (2009). Analysis of C1q polymorphisms suggests association with systemic lupus erythematosus, serum C1q and CH50 levels and disease severity. Ann. Rheum. Dis. 68, 715–720. 10.1136/ard.2007.085688 18504288

[B29] Mejia-Vilet J. M. Parikh S. V. Song H. Fadda P. Shapiro J. P. Ayoub I. (2019). Immune gene expression in kidney biopsies of lupus nephritis patients at diagnosis and at renal flare. Nephrol. Dial. Transplant. 34, 1197–1206. 10.1093/ndt/gfy125 29800348 PMC7967887

[B30] Mitchell D. A. Pickering M. C. Warren J. Fossati-Jimack L. Cortes-Hernandez J. Cook H. T. (2002). C1q deficiency and autoimmunity: the effects of genetic background on disease expression. J. Immunol. 168, 2538–2543. 10.4049/jimmunol.168.5.2538 11859149

[B46] Newman A. M. Steen C. B. Liu C. L. Gentles A. J. Chaudhuri A. A. Scherer F. (2019). Determining cell-type abundance and expression from bulk tissues with digital cytometry. Nat. Biotechnol. 37 (7), 773–782. 10.1038/s41587-019-0114-2 31061481 PMC6610714

[B31] Rhodes B. Hunnangkul S. Morris D. L. Hsaio L.-C. Cunninghame Graham D. S. Nitsch D. (2009). The heritability and genetics of complement C3 expression in UK SLE families. Genes Immun. 10, 525–530. 10.1038/gene.2009.23 19387462

[B32] Ritchie M. E. Phipson B. Wu D. Hu Y. Law C. W. Shi W. (2015). Limma powers differential expression analyses for RNA-sequencing and microarray studies. Nucleic Acids Res. 43, e47. 10.1093/nar/gkv007 25605792 PMC4402510

[B33] Robin X. Turck N. Hainard A. Tiberti N. Lisacek F. Sanchez J.-C. (2011). pROC: an open-source package for R and S+ to analyze and compare ROC curves. BMC Bioinforma. 12, 77. 10.1186/1471-2105-12-77 PMC3068975 21414208

[B34] Salloum R. Niewold T. B. (2011). Interferon regulatory factors in human lupus pathogenesis. Transl. Res. 157, 326–331. 10.1016/j.trsl.2011.01.006 21575916 PMC3096827

[B35] Sebastiani G. D. Galeazzi M. Tincani A. Scorza R. Mathieu A. Passiu G. (2003). HLA-DPB1 alleles association of anticardiolipin and anti-beta2GPI antibodies in a large series of European patients with systemic lupus erythematosus. Lupus 12, 560–563. 10.1191/0961203303lu402oa 12892399

[B36] Shannon P. Markiel A. Ozier O. Baliga N. S. Wang J. T. Ramage D. (2003). Cytoscape: a software environment for integrated models of biomolecular interaction networks. Genome Res. 13, 2498–2504. 10.1101/gr.1239303 14597658 PMC403769

[B37] Sheng Y. Xu J. Wu Y. Zuo X. Gao J. Lin Y. (2015). Association analyses confirm five susceptibility loci for systemic lupus erythematosus in the Han Chinese population. Arthritis Res. Ther. 17, 85. 10.1186/s13075-015-0602-9 25890262 PMC4404072

[B38] Sun-Tan Ç. Özgür T. T. Kılınç G. Topaloğlu R. Gököz Ö. Ersoy-Evans S. (2010). Hereditary C1q deficiency: a new family with C1qA deficiency. Turkish J. Pediatr. 52, 184–186. 20560256

[B39] Svetnik V. Liaw A. Tong C. Culberson J. C. Sheridan R. P. Feuston B. P. (2003). Random forest: a classification and regression tool for compound classification and qsar modeling. J. Chem. Inf. Comput. Sci. 43, 1947–1958. 10.1021/ci034160g 14632445

[B40] Szklarczyk D. Gable A. L. Lyon D. Junge A. Wyder S. Huerta-Cepas J. (2019). STRING v11: protein–protein association networks with increased coverage, supporting functional discovery in genome-wide experimental datasets. Nucleic Acids Res. 47, D607–D613. 10.1093/nar/gky1131 30476243 PMC6323986

[B41] Udhaya Kumar S. Thirumal Kumar D. Siva R. George Priya Doss C. Younes S. Younes N. (2020). Dysregulation of signaling pathways due to differentially expressed genes from the B-cell transcriptomes of systemic lupus erythematosus patients – a bioinformatics approach. Front. Bioeng. Biotechnol. 8, 276. 10.3389/fbioe.2020.00276 32426333 PMC7203449

[B42] Wu T. Hu E. Xu S. Chen M. Guo P. Dai Z. (2021). clusterProfiler 4.0: a universal enrichment tool for interpreting omics data. Innovation 2, 100141. 10.1016/j.xinn.2021.100141 34557778 PMC8454663

[B43] Wu W.-J. Tan Y. Liu X.-L. Yu F. Zhao M.-H. (2020). C1q A08 is a half-cryptic epitope of anti-C1q A08 antibodies in lupus nephritis and important for the activation of complement classical pathway. Front. Immunol. 11, 848. 10.3389/fimmu.2020.00848 32536911 PMC7267003

[B44] Yang H. Li H. (2019). *CD36* identified by weighted gene co-expression network analysis as a hub candidate gene in lupus nephritis. PeerJ 7, e7722. 10.7717/peerj.7722 31592160 PMC6777479

[B45] Yap D. Y. H. Chan T. M. (2019). B cell abnormalities in systemic lupus erythematosus and lupus nephritis—role in pathogenesis and effect of immunosuppressive treatments. IJMS 20, 6231. 10.3390/ijms20246231 31835612 PMC6940927

