# Supplementary material for: Comprehensive analysis of IRF8-related genes and immune characteristics in lupus nephritis
Source: Front Pharmacol. 2024 Dec 9;15:1468323. doi: 10.3389/fphar.2024.1468323 (PMC11663682; doi:10.3389/fphar.2024.1468323)
Supplement: Supplementary file 2 [file Table1.DOC]

**Supplementary Table 1. Murine primer sequences**

| Gene | Sense primer | Antisense primer |
| --- | --- | --- |
| Gapdh | AGGTCGGTGTGAACGGATTTG | TGTAGACCATGTAGTTGAGGTCA |
| Irf8 | CGGGGCTGATCTGGGAAAAT | CACAGCGTAACCTCGTCTTC |
| C1qa | AGTCCATACCAGAACCACACG | CCACTTGGAGATCACTTGGAA |
| Itgb2 | ACACCTCTTGTGCCGAGTGCCT | ATCCAACAGCCTTCCGAGTCCCT |
| Tollip | CCACATCCTCTTGTTTAGTACCA | ATCTCCTTCGCTTGACTCTG |
